# Patient-derived organoids and high grade serous ovarian cancer: from disease modeling to personalized medicine

**DOI:** 10.1186/s13046-021-01917-7

**Published:** 2021-03-31

**Authors:** Camilla Nero, Giuseppe Vizzielli, Domenica Lorusso, Eleonora Cesari, Gennaro Daniele, Matteo Loverro, Giovanni Scambia, Claudio Sette

**Affiliations:** 1 Fondazione Policlinico Universitario A. Gemelli IRCCS, L.go Agostino gemelli, 8, 00168 Roma, Italy; 2 Dipartimento di Scienze della vita e sanità pubblica, Università Cattolica del Sacro Cuore, Roma, Italy; 3 Dipartimento di Neuroscienze, Sezione di Anatomia Umana, Università Cattolica del Sacro Cuore, Roma, Italy

**Keywords:** Ovarian cancer, Organoids, Target therapy, 3D cultures, Drug screening

## Abstract

**Background:**

High grade serous ovarian cancer (HGSOC) is among the deadliest human cancers and its prognosis remains extremely poor. Tumor heterogeneity and rapid acquisition of resistance to conventional chemotherapeutic approaches strongly contribute to poor outcome of patients. The clinical landscape of HGSOC has been radically transformed since the advent of targeted therapies in the last decade. Nevertheless, the lack of predictive biomarkers informing on the differential clinical benefit in select subgroups, and allowing patient-centric approaches, currently limits the efficacy of these novel therapies. Thus, rational selection of the best possible treatment for each patient represents a clinical priority in order to improve outcome, while limiting undesirable effects.

**Main body:**

In this review, we describe the state of the art and the unmet needs in HGSOC management, illustrate the treatment options that are available and the biomarkers that are currently employed to orient clinical decisions. We also describe the ongoing clinical trials that are testing new therapeutic approaches for HGSOC. Next, we introduce the organoid technology as a promising, expanding strategy to study cancer and to develop personalized therapeutic approaches. In particular, we discuss recent studies that have characterized the translational potential of Patient’s Derived Organoids (PDOs) to inform on drug sensitivity of HGSOC patients.

**Conclusions:**

PDOs can predict the response of patients to treatments and may therefore guide therapeutic decisions. Although preliminary results appear encouraging, organoids still need to be generated and expanded efficiently to enable drug screening in a clinically meaningful time window. A new generation of clinical trials based on the organoid technology should guarantee tailored approaches to ovarian cancer management, as it is now clear that the one-size-fits-all approach cannot lead to efficient and meaningful therapeutic advancements.

## Background

The overall survival of patients with ovarian cancer (OC) had not substantially changed for several decades, mainly due to the lack of early diagnosis coupled with frequent acquisition of resistance to therapeutic treatments [1, 2]. Nevertheless, recent progress in traditional and novel therapeutic strategies have led to a significant improvement in patient’s outcome especially for the high grade serous histotype (HGSOC), which is the most common and most malignant among the OC subtypes [3–5]. In particular, several treatment options have become available in the past 5 years, both for frontline and recurrent disease settings.

Decisions regarding the best treatment option for each individual patient do not always have an obvious “optimal choice”, due to a number of variables that are not always easy to decipher. First, both the type and the timing of treatment(s) have to be considered. Moreover, while algorithms based on patient’s characteristics and biological factors are necessary, they are not diagnostically exhaustive. Lastly, the identification of successful therapies is also hampered by the high level of complexity and genetic heterogeneity existing even within single tumor types. With more therapeutic options being available, the identification of biological markers and/or experimental models that are able to predict treatment response has progressively become a clinical priority.

In this scenario, an emerging technology that holds promise of significantly impacting the clinical management of patients in the near future is represented by patient-derived Organoids (PDOs). Indeed, elegant studies carried out in the past 2–3 years have clearly indicated that HGSOC PDOs have the potential to faithfully reproduce many of the challenging characteristics of the tumor from which they derive in a reasonable time frame and at sustainable costs [6–10]. This technology may offer the possibility of attaining truly personalized drug-based therapy. On this basis, it is conceivable that PDOs could be introduced as a clinical test to guide the selection of therapeutic treatments and to improve management of HGSOC patients in the near future.

## State of the art and unmet needs in ovarian cancer clinical management

### Treatment options and predictive biomarkers

**Fig. 1:**
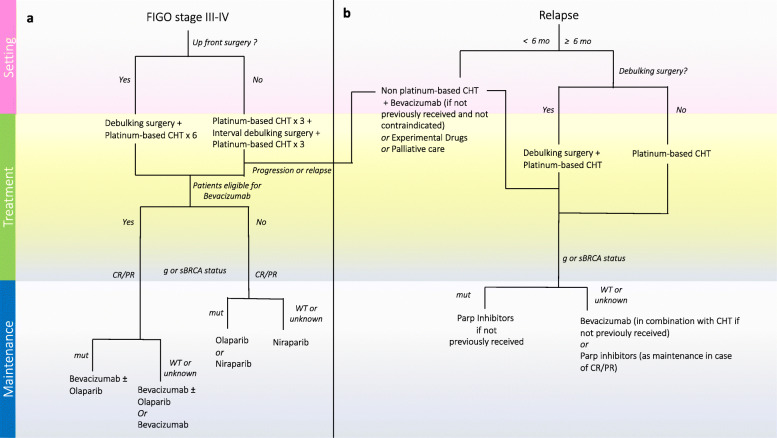
HGSOC treatment options according to ESMO and NCCN recommendations in front line **a** and recurrent **b** clinical settings

The current treatment options for advanced stage and recurrent HGSOC according to ESMO and NCCN recommendations [11, 12] are summarized in Fig. 1.

**Fig. 2:**
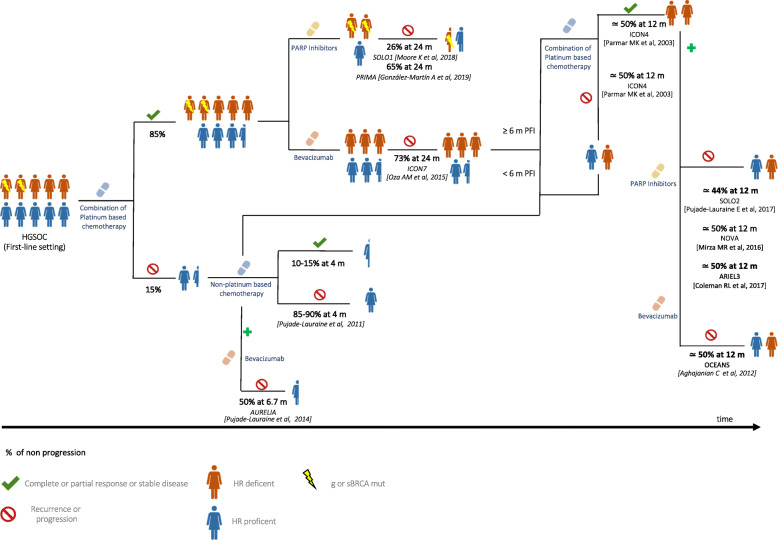
Efficacy of the currently available treatments in HGSOC. Data are expressed as the median rate of freedom from disease progression and from death at a certain interval time

Treating HGSOC has become an increasingly complex chess game in which clinicians should be fully aware of the consequences that each move implies. Identifying tumors that will respond to the available therapies in that precise moment of the disease is an emerging unmet need that will progressively overcome every other traditional debate. In this regard, the efficacy of current available treatments measured by progression or relapse rate is represented in Fig. 2.

The standard of care for newly diagnosed HGSOC patients consists of primary debulking surgery (PDS) and platinum-based chemotherapy. The amount of residual disease after surgery remains a key prognostic variable supporting the role of PDS with maximal debulking of tumour [13, 14]. Although desirable, optimal or suboptimal (less than 1 cm in maximum diameter of residual tumour) surgeries are only achieved in 25–40% of cases undergoing PDS worldwide [15]. Thus, neoadjuvant chemotherapy (NACT) followed by interval debulking surgery (IDS) has been tested as an alternative option. At least four randomized controlled trials were conducted to compare NACT versus PDS in the past years. Although debates on this topic continue, NACT has become an established practice of care in patients with high tumor load or severe comorbidities [16–19].

The combination of carboplatin and paclitaxel is the ‘backbone’ of the chemotherapeutic treatment for advanced HGSOC [20–22]. While most HGSOC patients show a good response to the conventional platinum-based chemotherapy, ~ 15% of women experience primary resistance to these treatments [20–24]. To date, there are no validated predictive markers of primary platinum refractory or resistant disease. Although limited and biologically unfounded, the time since the last platinum chemotherapy currently defines resistance (≤ 6 months) or sensitivity (> 6 months) to the treatment in HGSOC patients that experience recurrent disease [25, 26].

Subsequent randomized trials have been designed adding a third drug, either in combination or in maintenance. These trials lead to the approval of Bevacizumab and two types of poly-ADP-ribose polymerase (PARP) inhibitors (Olaparib and Niraparib) [26–29]. Bevacizumab is a humanized monoclonal antibody against vascular endothelial growth factor A (VEGF-A). Unfortunately, no molecular biomarkers can faithfully predict its benefit in patients. Indeed, neither angiogenic markers (i.e. CD31, microvessel density and tumor VEGF-A levels) nor predictive signatures based on combinations of biomarkers (i.e. mesothelin, FLT4, alpha-1 acid glycoprotein and CA125 or Ang1 and Tie2) were prospectively validated in large clinical trials and, therefore, were not introduced into clinical practice [30, 31]. Currently, only clinical biomarkers, including stage, debulking status, residual tumor and presence of ascites, are used to select patients for first line treatment with Bevacizumab. Olaparib and Niraparib are inhibitors of the PARP-1 and -2 enzymes and are able to selectively kill cancer cells that are defective for the BRCA 1 and 2 tumor suppressors. PARP1/2, like BRCA1/2, are involved in the DNA damage response pathway that promotes homologous recombination (HR) and repair of the DNA lesions. Thus, concomitant inhibition of this pathway by *BRCA1/2* mutations and PARP inhibitors (PARPi) results in synthetic lethality of cancer cells [32, 33]. However, although mutations in *BRCA1/2* genes represent the strongest hallmark of sensitivity to PARPi, up to 40% of HGSOC patients bearing these mutations fail to respond to treatment [32]. On the other hand, tumors harboring other mutations that impair the HR pathway exhibited remarkable responses to PARPi [32–35]. Thus, direct evaluation of HR proficiency may help stratify more accurately the HGSOC patients who would benefit of treatment with PARPi. However, current assays of HR proficiency are believed to be unsuitable to exclude patients from PARPi therapy, at least in second line platinum sensitive relapse, in which platinum sensitivity is still considered the best predictor of response to PARPi [11]. The evaluation of genome-wide loss of heterozygosity (LOH) or genomics scars (a score based on telomeric imbalance, LOH and large-scale rearrangements) could indirectly assess HR deficiency status, including also patients whose tumors harbor HR-impairing mutations different from those in the *BRCA1/2* genes. However, attempts to use these tests as predictive markers in the maintenance setting were not completely successful and the tests were not conclusive in up to 20% of patients due to technical issues [36, 37]. Moreover, reversion of HR deficiency, which may occur upon development of resistance to platinum and to PARPi and likely contributes to clinical drug resistance, is a major limitation of current HR assays [38, 39].

Overall, targeted therapies produced a paradigm shift in the treatment of HGSOC, transforming it into a chronic condition. Up to 80% of HGSOC patients relapses within 24 months and treatment options at that time are also conditioned by first line treatments [40]. The best treatment sequence or combination challenges clinicians as much as the optimal selection of patients for each treatment. Differently from Bevacizumab whose efficacy beyond progression has been clearly reported, it is at present unclear whether PARPi maintain efficacy in a second treatment and if they can be used also in patients who have previously received PARPi [41, 42].

### Drugs under investigation

Most ongoing clinical trials for HGSOC are focused on immune check point inhibitors which are mainly based on inhibiting the PD-1/PD-L1 pathway. The efficacy of these agents depends on many factors, including PD-L1 expression, abundance of tumor infiltrating lymphocytes (TILs), neoantigen load and tumor mutational burden [43, 44]. Initial over-optimism about these agents in HGSOC treatment has been tempered by the disappointing results emerged in clinical trials [45–50].

In particular, in recurrent patients, immune check point inhibitors failed to demonstrate any relevant benefit both as single agent (KEYNOTE-100, single arm trial on Pembrolizumab) and in combination with chemotherapy (JAVELIN 200, randomized trial pegylated liposomal doxorubicin and Avelumab vs pegylated liposomal doxorubicin single agent vs Avelumab single agent; MK-3475, single arm trial on Pembrolizumab in combination with weekly paclitaxel) or with antiangiogenetic agents (NCT02873962, single arm on the combination of anti-PD1 Nivolumab with Bevacizumab) [46, 47, 49]. Clinical results were disappointing also in newly diagnosed patients. Indeed, the JAVELIN 100 phase III randomized trial, which tested Avelumab combined with platinum-based chemotherapy and as maintenance vs chemotherapy alone, was prematurely terminated for futility at the pre-planned interim analysis [48]. Likewise, the combination of Atezolizumab/Bevacizumab failed to demonstrate an improvement in progression free survival with respect to Bevacizumab alone in newly diagnosed HGSOC patients in the recently presented IMAGYN050 trial [50].

The combination of immune check point inhibitors with PARPi is supported by a strong rationale. Indeed, HR deficient (HRD) tumors are characterized by elevated PD-L1 expression and persistence of non-lethal DNA defects, which continuously stimulate innate immune cells to release pro-inflammatory substances. This tumor microenvironment probably induces the switch from a Th1-mediated immunity to chronic inflammation and immunosuppression [51]. PARPi, by triggering catastrophic DNA damage, especially in HRD cells, could restore a productive Th1 immune response and reset the tumor microenvironment [52]. In line with this hypothesis, it was reported that in mouse models bearing mutations in the *Brca1/2* genes, PARPi increased the mutational tumor load, promoted the recruitment of TILs and activated the interferon-mediated pathway by synergizing with immune check point inhibitors [52].

Lastly, the redundant nature of immune control and the cross talk between signaling pathways strongly support the blockade of multiple immune checkpoints as a strategy to improve the efficacy of anti-PD1-PDL1 therapy and to overcome resistance [53]. On this basis, many clinical trials are currently verifying both hypotheses in first line and recurrent clinical settings [54–62].

### Patient-derived organoids

Human organoids are stem cell-derived three-dimensional (3D) culture systems exhibiting interesting perspectives in both basic and translational research [6, 63, 64]. Organoid cultures have been generated from almost all endoderm-derived tissues and were demonstrated to faithfully recapitulate the features of the tissue of origin [63]. Furthermore, these 3D cultures can also be used to propagate tumor tissues, thus providing an in vitro model for the study of human cancers [6, 64]. Tumor organoid lines have been obtained from many types of epithelial cancers, such as lung, esophageal, bladder, endometrial, ovarian, renal, colorectal, gastrointestinal, pancreatic, prostate, breast and liver cancer [6, 63, 64], and were also recently cultured from glioblastoma tissues [65, 66].

An important feature of PDOs is that they recapitulate the cellular heterogeneity that characterizes the tumor from which they are derived, thus allowing high quality modeling of human carcinogenesis [6, 63, 64]. PDOs can be expanded for long-term, cryopreserved in biobanks and efficiently recovered after thawing [6, 63–66]. Furthermore, the relatively simple culture conditions and limited costs required to maintain them, make PDOs excellent models also for in vitro drug screening [6]. Indeed, several studies in the past three years have clearly shown that drug responses in PDOs summarize patient’s responses in the clinic [6, 63–66].

The success rate of initiation, time of establishment, ease of maintenance and growth rates of PDOs vary considerably, depending on the type of cancer and on the percentage of proliferating cells in the specific biopsy. Nevertheless, once established the PDOs display several advantages with respect to other cancer model systems. Compared to the classical 2D cancer cell lines, organoids are more difficult to operate and more expensive to maintain. However, they represent more reliably the pathological features of the tumor, as PDOs maintain genetic stability and tumor heterogeneity [6, 8–10, 67–74], which instead are lost during the long-term selection required to establish 2D tumor cell lines. In comparison with patient-derived xenografts (PDXs) or genetically engineered animal models, PDOs are less time consuming, less expensive and more appropriate for high-throughput drug screening. Furthermore, PDOs are usually obtained with higher success rate than PDXs and can also be formed from non-transformed cell cultures or preinvasive cancer models [6, 8–10, 67–74]. Lastly, with respect to spheroids, which are clusters of proliferating cells that assemble in 3D sphere-like structures floating in the culture medium [75], PDOs offer a better representation of the architecture of the tumor, as they assemble onto a reconstituted matrix that resembles the basal lamina of epithelia and recapitulate the cellular heterogeneity of the tumor mass. Nevertheless, one limitation of PDOs is currently represented by the lack of reliable protocols capable to faithfully reproduce the tumor microenvironment, which comprises the stroma, immune cells and blood vessels [6, 8–10, 67–74]. However, the recent development of organoids cocultured with tumor microenvironment components holds promise for the possibility to evaluate complex features of tumors in the near future [76, 77]. Short term PDO cultures were recently shown to maintain tumor infiltrating immune cells and have been successfully employed for comparative analyses of immune checkpoint therapies responses [77]. Moreover, in cancers characterized by a high tumor mutational burden, co-culture of PDOs with peripheral blood lymphocytes generated CD8+ T cell clones specifically reactive against neoplastic cells, thus potentially valuable for adoptive cell transplantation [78]. Another current limitation to application of PDO platforms to clinical trials is the relatively limited information regarding their predictive value in terms of response to treatments. Nevertheless, eighteen clinical trials including various cancer types are currently evaluating this issue, by testing the consistency and accuracy of PDOs to predict the clinical efficacy of anti-cancer drugs (NCT03979170, NCT04279509, NCT03577808, NCT04261192, NCT03925233, NCT03544255, NCT03453307, NCT03952793, NCT03655015, NCT03990675, NCT04371198, NCT04342286, NCT04777604, NCT04736043, NCT03500068, NCT04278326, NCT03890614). These issues are crucial because, in spite of the great improvement that PDOs have brought to preclinical cancer research, several challenges still need to be addressed before they can exert a concrete impact in clinical advance. For instance, validation of short-term organoids as tools that faithfully mimic the tumor microenvironment elements (i.e. fibroblasts, immune cells, vascular populations) is necessary in order to use PDOs as accurate platform for high-throughput drug and immunotherapy screens on single patients’ cancer sample. Furthermore, reliability of the drug response of PDOs as prognostic factor needs to be evaluated on large cohorts before it can enter in clinical routine at single patient level. It is conceivable that once these limitations are overcome, PDOs will be routinely used for a new generation of personalized clinical trials.

### Ovarian cancer PDOs

**Fig. 3:**
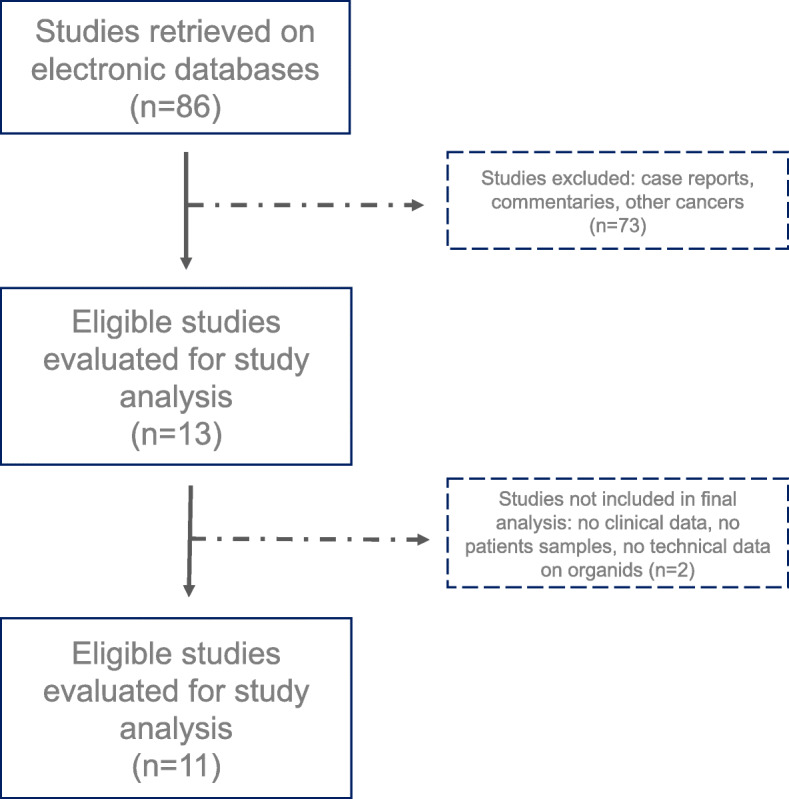
Consort Diagram

To investigate the state of the art in the research employing PDOs from OC tissues, we carried out a systematic review and meta-analysis (PRISMA) of the literature indexed in PubMed, MEDLINE and EMBASE electronic databases using the following terms: ‘organoids’ AND ‘ovarian cancer’. All types of articles were included, with the exception of case reports and commentaries or studies that did not fully report clinical and technical data on organoids features, establishment and maintenance (see the consort diagram represented in Fig. 3).

Several protocols to obtain PDO cultures from OC have been reported in the past three years [6, 8–10, 67–74]. The main experimental conditions and key findings of these studies are summarized in Table 1. Overall, the published data indicate the efficient derivation (success rate, ranging from 60 to > 90%) of a wide variety of OC subtypes (high grade and low grade serous carcinoma, carcinosarcoma, clear cell carcinoma, endometrioid carcinoma, mucinous and serous borderline tumor, malignant Brenner tumor) [69]. The timing required for the establishment of OC PDOs in culture varied significantly between cases, ranging from one to four weeks [6, 8–10, 67–74]. Importantly, it was possible to obtain PDOs from both the primary tumor and the metastatic lesions, therefore deriving multiple organoid lines from individual patients, a strategy that allows to also address the heterogeneity of the tumor.

**Table 1:** Key details and findings of studies on OC PDOs

	Pauli 2017* [67]	Hill 2018 [8]	Phan 2019 [68]	Kopper 2019 [9]	Maru 2019 [69]	Hoffman 2020 [70]	Sun 2020 [71]	Nanki 2020 [72]	Chen 2020 [73]	Maenhoudt 2020 [74]	de Witte 2020 [10]
Patients materials (n of patients)	Fresh Tissue	- Fresh tissue - Pleural effusion	Fresh tissue	Fresh tissue - Pleural effusion - Ascites	Fresh tissue	Fresh tissue	Fresh tissue	Fresh tissue	-Ascites -Pleural effusion	Fresh tissue	- Fresh tissue - Ascites
Origin of biological material (n of samples)	Ovary	- Omentum (15) - Ovary (12) - Pleural effusion (1) - Mesentery (5) - Diaphragm (1)	- Ovary (1) - Peritoneum (1)	- Ovary (28) - Peritoneum (4) - Omentum (9) - Pleural effusion (2) - Lymph node (1) - Diaphragm (3) - Bowel (1) - Uterus (1) - Abdominal wall (3) - Ascites (4)	NA	- Peritoneum (10) - Omentum (5)	NA	Ovary	Ascites (8) -Pleural effusion (1)	- Omentum (3) - Ovary (3) - Rectum (1) - NA (24)	- Omentum (7) - Ovary/adnexa (19) - Peritoneum (2) - Lymph node (1) - Ascites (3) - Uterus (2) - Abdominal wall (2)
Clinical setting (n of samples per patient)	NA	- Untreated (10) - NACT (12) - Recurrent (2)	- Untreated (2) - NACT (2)	- Untreated (17) - NACT (9) - Recurrent (7)	NA	- Untreated (12) - NACT (1)	- Untreated (4) - Treated (6)	- Untreated (6)*** - NACT (1)***	- Untreated (3)*** - Treated (11)***	- Untreated (9) - NACT (20) - Recurrent (2)	- Untreated (21) - NACT (9) - Recurrent (6)
Histological types (n)	S (1)	HGS (22) LGS (1)	HGM (1) HGS (1) HGP (1) CS (1)	HGS (2) E (2) HG (2) LGS (14) CC (1) SBT (4) M (5) MBT (7)	E (4) BBT (1) HGS (4) MBT (1) MCB (2) SBT (2) M (1)	HGS (45)	S (10)	HGS (10) CC (10) E (5) MBT (3) Others ** (7)	HGS (5) HGP (1)	HGS (22) LGS (2) MMMT (1) M (1) CC (1)	HGS (11) E (1) HG (2) LGS (4) CC (1) S/MBT (2) M (3)
Number of patients/number of organoids	1/1	23/33	4/4	32/56	15/9	13/15	10/10	35/28	6/14	27/12	23/36
Overall success rate (%)	100	80–90%	100%	65%	60%	30%	n.s.	80%	n.s.	44%	n.s.
Onset of organoid formation (days)	n.s	7–14 days	n.s.	3–14 days	n.s.	n.s.	n.s.	7–21 days	3–4 days	n.s.	20 days
Expansion	5 passages	2 passages	n.s.	3–31 passages	n.s.	6–26 passages	n.s.	≥ 4 passages	n.s.	1–2 passages	n.s.
Extracellular matrix	Matrigel	Matrigel	Matrigel or Cultrex BME	Matrigel	Matrigel	Matrigel	Matrigel	Matrigel	Cultrex BME	Matrigel	Matrigel
Culturing Medium	-Advanced DMEM -Glutamax 1X -B27 −100 U/ml Penicillin −100μg/ml Streptomycin -Primocin 100μg/ml - N-Acetylcysteine 1.25 mM - Mouse Recombinant EGF 50 ng/mL - Human Recombinant FGF-10 20 ng/mL - Recombinant Human FGF-basic 1 ng/mL - Y-27632 10uM - A-83-01500 nM - SB202190 10uM - Nicotinamide 10 mM - PGE2 1uM - Noggin 50 mL - R-Spondin 25 mL	-Advanced DMEM/F12, − 1% penicillin streptomycin- Glutamax 1X -1% HEPES − 100 ng/mL R-spondin 1 -100 ng/mL Noggin −50 ng/mL EGF − 10 ng/mL FGF-10 − 10 ng/mL FGF2 − 1× B27 − 10 mM Nicotinamide − 1.25 mM N-acetylcysteine -1uM Prostaglandin E2 -10uM SB202190 - 500 nm A83–01	PrEGM medium or Mammocult	- ADF ± 25% conditioned human Wnt3A medium − 25% conditioned human RSPO1 medium − 12 mM HEPES − 1% GlutaMAX − 2% B27 − 1% N2 − 10 ng ml^− 1^ human EGF − 100 ng ml^− 1^ human noggin − 100 ng ml^− 1^ human FGF10–1 mM nicotinamide − 9 μM ROCK inhibitor − 0.5 μM TGF-β R Kinase Inhibitor IV -hydrocortison -forskolin -heregulinβ-1	-advanced DMEM/F12 - 50 ng/ml human EGF − 250 ng/ml R-spondin1 − 100 ng/ml Noggin - 10 μMY27632 − 1 μM Jagged-1 - L-glutamine solution -penicillin/ Streptomycin and amphotericin B suspension	-Advanced DMEM/F12 1X - penicillin streptomycin 100 U·ml^− 1^ / 100 mg·ml^− 1^ - 10 mM HEPES - GlutaMax 100x 1X - Nicotinamide 1 mM - N2 supplement 1X - B27 supplement 1X - SB431542 0.5 μM - - R-Spondin 1, mouse 25% - EGF 10 ng·ml^− 1^ - Y-27632 9 μM	n.s.	- Advanced DMEM/F12 − 2 mM HEPES - 1 × GlutaMAX-I -1X B27 supplement − 10 nM Leu15-Gastrin I - 1 mM N-acetylcystein − 100 ng/mL recombinant human IGF-1 − 50 ng/mL recombinant human FGF-2 - 20% Afamin/Wnt3a CM - 1 μg/ mL humanR-spondin - 100 ng/mL Noggin − 500 nM A-83-01 − 200 U/mL penicillin/streptomycin − 10 μM Y-27632	-DMEM/ F12 − 10% R-spondin1 - 2% B27 supplement - 10 mM HEPES − 1% Glutamax − 1.25 mM N-acetyl cysteine − 100 μg/mL Primocin - 1% Antibiotic- Antimycotic - 1 mM nicotinamide − 0.5 μM A 83–01 − 5 nM Neuregulin 1 - 5 ng/mL FGF-7 - 20 ng/mL FGF-10 − 100 ng/mL Noggin - 5 ng/mL EGF - 0.5 μM SB 202190 - 5 μM Y-27632	- Y27632 10 μM -DMEM/F12 -L-glutamine 1X - Pen/Strep 1X - A83–01 0.5 μM -Nicotinamide 1 or 5 mM -N2 1X B27 minus vitamin A N-acetylcysteine 1X - 17-β Estradiol 1.25 mM - p38i 1 or 10 μM - EGF 50 ng/ml ± bFGF 2 ng/ml ± FGF10 10 ng/ml -Noggin (rec or CM) 10% or 100 ng/mL - RSPO1 (rec or CM) 25% or 50 ng/mL ±IGF1 20 ng/mL ±HGF 10 ng/mL ±NRG1 50 ng/mL	- ADF ± 25% conditioned human Wnt3A medium − 25% conditioned human RSPO1 medium − 12 mM HEPES − 1% GlutaMAX − 2% B27 − 1% N2 − 10 ng ml^− 1^ human EGF − 100 ng ml^− 1^ human noggin − 100 ng ml^− 1^ human FGF10 − 1 mM nicotinamide − 9 μM ROCK inhibitor − 0.5 μM TGF-β R Kinase Inhibitor IV -hydrocortison -forskolin -heregulinβ-1
Genomic characterization	WES (at passage 5)	WES	Not performed	WGS	409-gene panel (on 3 out of 9 PDOs)	121-gene panel	RNA-Seq analysis	1053-gene panel (at median passage 4)	RNA-Seq analysis	WGS	WGS
Concordance rate	86% Allele-specific copy number, high concordance in ploidy and genomic burden	> 98% in somatic mutation, allelic imbalance and copy number variantions	n.s	High in in somatic mutation, allelic imbalance and copy number variations	High in somatic mutations	High in somatic mutations	Not performed	Median 59.1% of gene variants were shared; high concordance in copy number variations	n.s	High in in somatic mutation and copy number variations	67% of single nucleotide variants, comparable copy-number states
Drug screening	Not performed	Carboplatin Olaparib Prexasertib VE-822	Seliciclib Milciclib PHA-793887 PHA-767491 BS-181 HCl BMS-265246 Flavopiridol HCl BMS-387032 AT7519 Dinaciclib Degrasyn R547 Alvocidib AZD5438 JNJ-7706621 THZ1 Palbociclib SNS-032 WZ3146 WZ8040 IMD0354 PD184352 AZD8330 Omipalisib BGT226 Quizartinib BGT226 Degrasyn Lapatinib Ditosylate Sorafenib Tosylate WZ8040 Lapatinib CHIR-124 CUDC-907 CUDC-101 NVP-AEW541 PHA-665752 GSK690693	Paclitaxel Carboplatin Alpelisib Pictilisib MK2206 AZD8055 Niraparib Adavosertib Gemcitabine	Paclitaxel Cisplatin	Carboplatin	Cisplatin	Cisplatin Carboplatin Paclitaxel Docetaxel Vinorelbine Eribulin Topotecan SN-38 Etoposide Doxorubicin Gemcitabine Tamoxifen Trabectedin Olaparib Vorinostat Belinostat Cediranib Pazopanib Sunitinib Everolimus Trametinib Gefitinib Lapatinib	Carboplatin Taxol Mocetinostat Trametinib LY294002 AZD5363 BBI503 MK-1775 Sorafenib APR-246 CB5083 Napabucasin	Paclitaxel Cisplatin Doxorubicin Gemcitabine	Carboplatin Paclitaxel Gemcitabine Olaparib Niraparib Rucaparib Afatinib Vemurafenib Flavopiridol Adavosertib Alpelisib Adavosertib Afatinib AZD8055 Pictilisib Cobimetinib

*S* Serous

*HGS* high grade serous

*LGS* Low grade serous

*HGM* High grade mixed type

*HGP* High grade peritoneal

*CS* carcinosarcoma

*E* endometrioid

*CC* Clear cell

*SBT* Serous borderline tumor

*M* mucinous

*MBT* Mucinous borderline tumor

*BBT* Borderline Brenner tumor

*MBT* Malignant brenner tumor

*MCB* Mucinous Cystoadenoma borderline

*MMMT* Malignant mixed mesonephric tumor

*NACT* neoadjuvant chemotherapy

*n.s.* not specified

* the study contains data from other cancer types

** dysgerminoma, thecoma, serous cystadenofibroma, carcinosarcoma, and fibroma

*** only referred to successfull organoids

A key feature of OC PDOs is to maintain the main hallmarks of the original tumor, including histological characteristics, biomarker expression, genomic profile and tumor heterogeneity [6, 8–10, 67–74]. In particular, OC PDOs were shown to faithfully recapitulate the genomic landscape of the original tumor and to be exploitable for functional profiling of DNA repair efficiency and response to therapeutic drugs. Indeed, when it was compared to the response of the patient, PDOs generally demonstrated similar features [6, 8–10, 67–74]. For instance, the HRD mutational signature of the PDO could predict sensitivity of both the organoid and the primary tumor to treatment with PARPi [8, 10]. Moreover, OC PDOs were successfully used to directly test HR proficiency by biological assays and this parameter was also in line with the sensitivity of the organoid to PARPi [9]. This observation suggests that OC PDOs could be used to assess the HRD status independently of the mutational signature, thus potentially uncovering also defects in genes and pathway not yet associated with HR.

Recent development in the organoid field have yielded culture conditions that allow long-term expansion of OC PDOs through slight modifications of the medium and growth factors utilized [70]. Together with the demonstration that OC PDOs can be cryopreserved [9, 70, 74], these results suggest that it will be possible to establish stable biobanks of PDOs with highly detailed features, to match the wide heterogeneity in tumor features that clinicians currently face. Such biobanks could be potentially employed for parallel screening of new drugs or drug combinations in a multicenter setting, thus favoring the employment of OC PDOs in clinical trials.

**Fig. 4:**
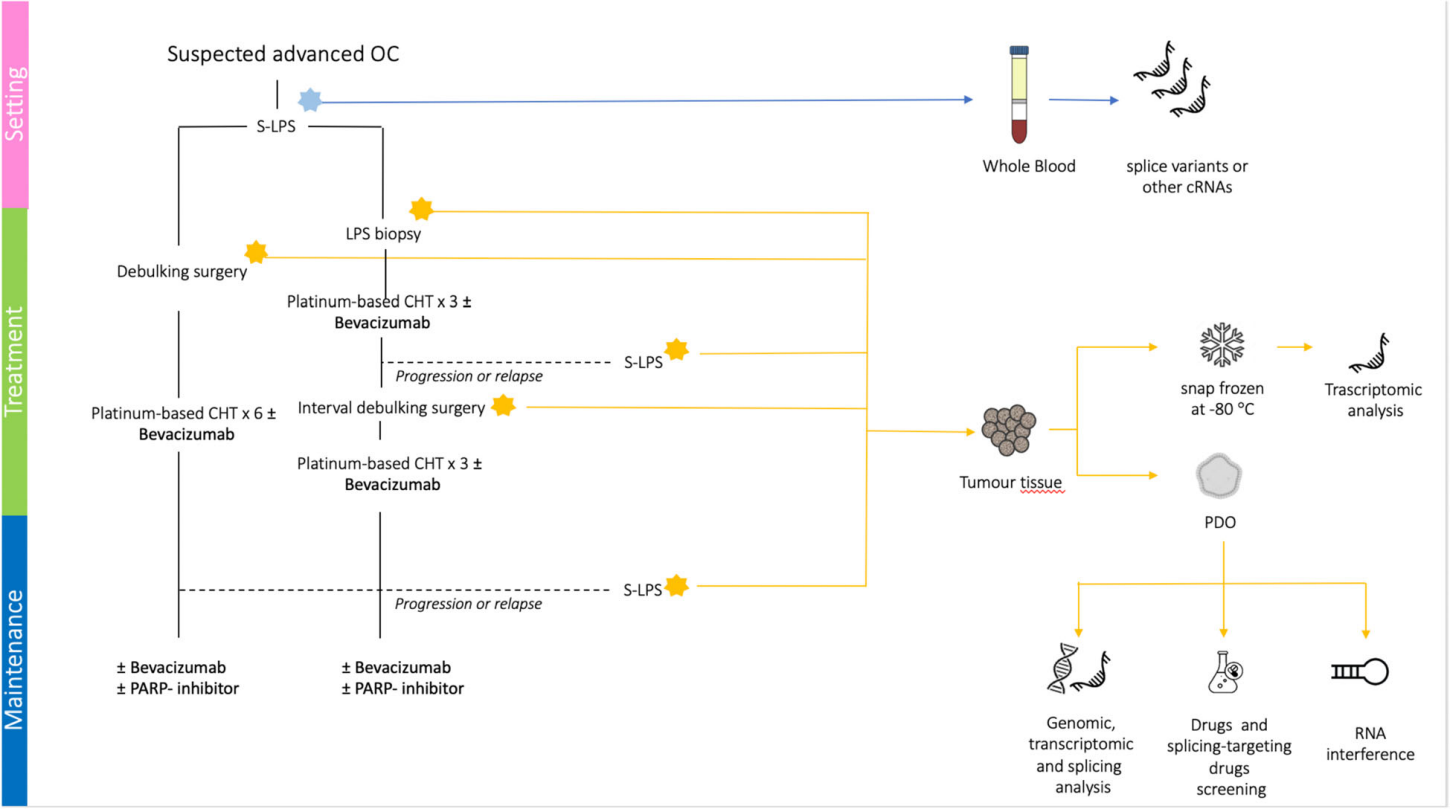
NCT04555473 trial design

Three clinical trials are currently ongoing to evaluate the role of PDOs in predicting the clinical efficacy of anti-cancer drugs in OC (NCT04279509, NCT04768270 and NCT04555473). The NCT04279509 trial is a single-centre study aimed at prospectively determine if high-throughput drug screen assays using PDOs can accurately select chemotherapeutic agents that result in objective response in patients with refractory solid tumours (head and neck squamous cell carcinoma, colorectal, breast and epithelial OC). NCT04768270 is a single-centre study aimed at verify whether PDOs can help guide precision treatments for OC patients. NCT04555473 is a longitudinal observational phase II study of the reliability of HGSOC PDOs as model for the patients’ response to treatments and it is conducted by our group. In this latter trial, PDOs are being established from both PDS and IDS cases preceded by NACT. Since organoids represent a model system comparable to PDXs, we tested the null hypothesis that the possibility of correctly identifying the drug-sensitivity could improve from 80% (as assessed by xenografts) to at least 95%. The first step was planned to include 7 patients; if 5 or more PDOs fail in the correct identification of drug-sensitivity compared to patients’ response, the trial will be terminated. If the trial proceeds to the second stage, a total of 43 patients will be studied. Considering a patient dropout of approximately 10%, the study was planned to enroll a minimum of 48 patients (see Fig. 4).

## Conclusions

HGSOC remains a devastating cancer for which new therapeutic strategies are urgently needed. In this scenario, PDOs represent reliable experimental models that can address several clinical challenges. However, current major bottlenecks regarding their use to support clinical decisions are related to efficacy, time, costs and accuracy in mimicking the overall cancer complexity.

In order to increase the efficacy, it will be crucial to standardize the procedures of tissue manipulation, the media and the growth factors required for each type of PDO. Such standardize guidelines would reduce the need of specialist skills and might promote the widespread usage of this technology in clinical settings. Major limits to significantly decrease the time for PDOs outgrowth are related to the quality of the biopsy. In particular, the possibility of having tissue fragments of sufficient size and enriched in proliferating cancer cells from laparoscopic biopsies or ultrasound guided biopsy would allow higher efficiency of PDO formation also from these surgical interventions. Costs will likely drop as technologies mature and protocols become standardized. However, median costs for PDO generation and maintenance are relatively high at the moment, thus representing a potential limitation for their application to routine management of patients. Lastly, the most limiting aspect of currently available PDOs is represented by the lack of tumor microenvironment. Large efforts are ongoing to include stromal, immune and vascular cells in these cultures, thus to represent more faithfully the disease. This is particularly relevant for HGSOC, where several targeted therapies involve immune checkpoints and angiogenic inhibitors. Nevertheless, mimicking the whole microenvironment characterizing the tumor is challenging, especially with respect to vascularization of the PDO. Improvement in these procedures will likely require substantial time and costs before the best and most reproducible conditions are set up.

**Fig. 5:**
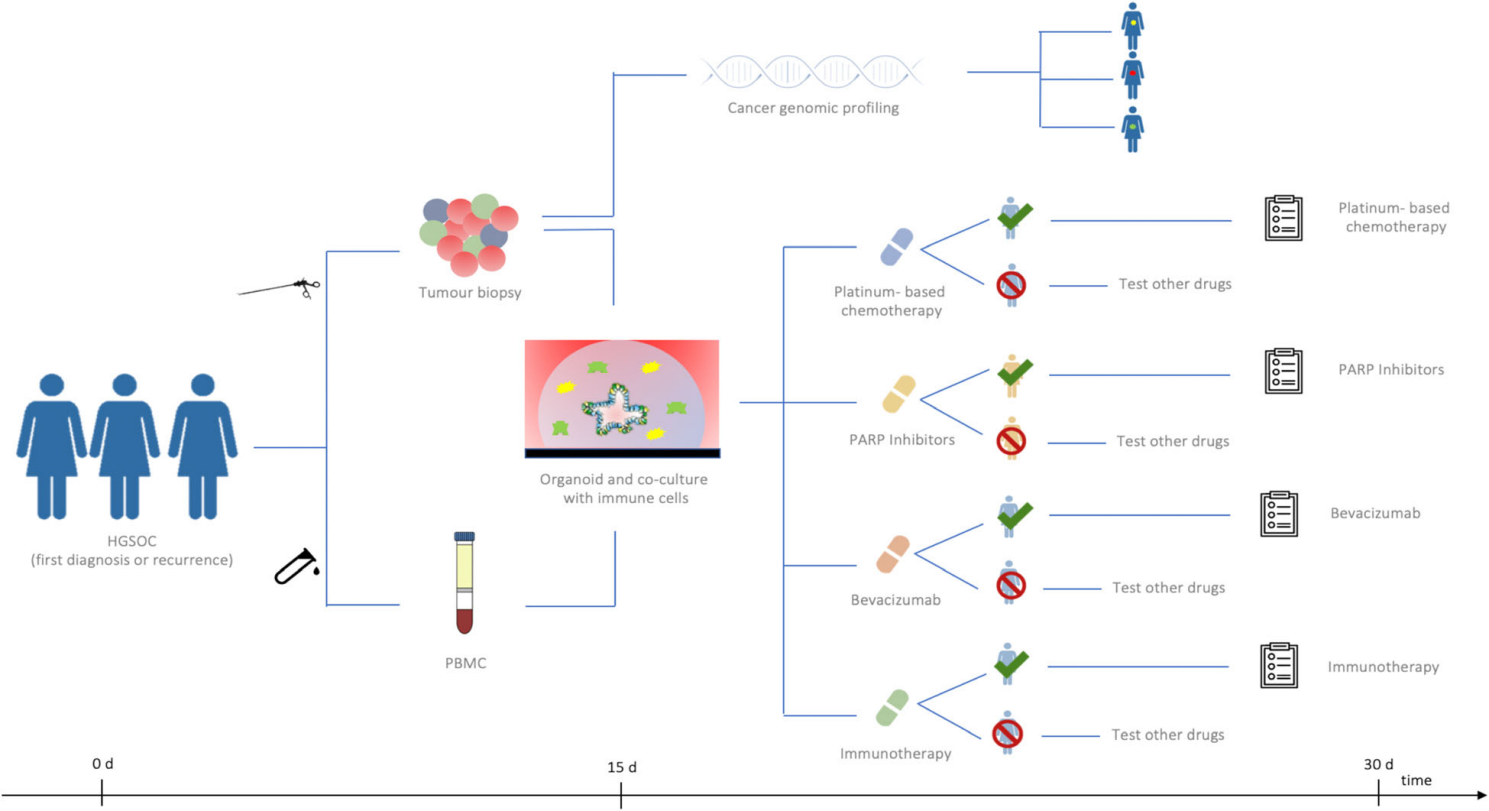
PDOs based future clinical trials

PDOs will certainly serve as a complement to other traditional models to study cancer, such as primary human tissues and animal models, which are currently the gold standard in biomedical research. However, once PDOs are improved and optimized, they may have unique characteristics to be introduced in future clinical trials as empirical predictor of patients’ response to therapies (see Fig. 5).

## Data Availability

Not applicable.

## Abbreviations

PDOs: Patients derived organoids
OC: Ovarian cancer
HGSOC: High grade serous ovarian cancer
PDS: Primary debulking surgery
IDS: Interval debulking surgery
NACT: Neoadjuvant chemotherapy
PDX: Patient-derived xenograft
LOH: Loss of heterozygosis
PARPi: Poly ADP ribose polymerase inhibitors
HRD: Homologous recombination deficiency
HR: Homologous recombination
PD-1: Programmed cell death protein 1
PD-L1: Programmed cell death protein 1 ligand
TILs: Tumor infiltrating lymphocytes
VEGF-A: Vascular endothelial growth factor A
3D: Three-dimensional

## References

[1] Bray F, Ferlay J, Soerjomataram I, Siegel RL, Torre LA, Jemal A (2018). Global cancer statistics 2018: GLOBOCAN estimates of incidence and mortality worldwide for 36 cancers in 185 countries. CA Cancer J Clin.

[2] Matulonis UA, Sood AK, Fallowfield L, Howitt BE, Sehouli J, Karlan BY (2016). Ovarian cancer. Nat Rev Dis Primers.

[3] Oza AM, Cook AD, Pfisterer J, Embleton A, Ledermann JA, Pujade-Lauraine E (2015). Standard chemotherapy with or without bevacizumab for women with newly di- agnosed ovarian cancer (ICON7): overall survival results of a phase 3 randomised trial. Lancet Oncol.

[4] Kaufman B, Shapira-Frommer R, Schmutzler RK, Audeh MW, Friedlander M, Balmaña J (2015). Olaparib monotherapy in patients with advanced cancer and a germline BRCA1/2 mutation. J Clin Oncol.

[5] van Driel WJ, Koole SN, Sikorska K, Schagen van Leeuwen JH, Schreuder HWR, Hermans RHM, de Hingh IHJT, van der Velden J, Arts HJ, Massuger LFAG, Aalbers AGJ, Verwaal VJ, Kieffer JM, Van de Vijver KK, van Tinteren H, Aaronson NK, Sonke GS. Hyperthermic Intraperitoneal Chemotherapy in Ovarian Cancer. N Engl J Med. 2018; 378(3): 230–240. doi: 10.1056/NEJMoa1708618. PMID: 29342393. 10.1056/NEJMoa1708618 29342393

[6] Tuveson D, Clevers H., Cancer modeling meets human organoid technology. Science. 2019; 364(6444): 952–955. doi: 10.1126/science.aaw6985. PMID: 31171691. 10.1126/science.aaw6985 31171691

[7] Lõhmussaar K, Boretto M, Clevers H. Human-derived model Systems in Gynecological Cancer Research. Trends Cancer 2020;6(12):1031–1043. doi: 10.1016/j.trecan.2020.07.007. Epub 2020 Aug 24. PMID: 32855097 10.1016/j.trecan.2020.07.007 32855097

[8] Hill SJ, Decker B, Roberts EA, Horowitz NS, Muto MG, Worley MJ, Feltmate CM, Nucci MR, Swisher EM, Nguyen H (2018). Prediction of DNA repair inhibitor response in short-term patient-derived ovarian Cancer Organoids. Cancer Discov..

[9] Kopper O, DeWitte CJ, Lõhmussaar K, ValleInclan JE, Hami N, Kester L, Balgobind AV, Korving J, Proost N, Begthel H (2019). An organoid platform for ovarian cancer captures intra- and interpatient heterogeneity. Nat Med.

[10] de Witte CJ, Espejo Valle-Inclan J, Hami N, Lõhmussaar K, Kopper O, Vreuls CPH, Jonges GN, van Diest P, Nguyen L, Clevers H, Kloosterman WP, Cuppen E, Snippert HJG, Zweemer RP, Witteveen PO, Stelloo E (2020). Patient-derived ovarian Cancer Organoids mimic clinical response and exhibit heterogeneous inter- and Intrapatient drug responses. Cell Rep.

[11] Colombo N, Sessa C, du Bois A, Ledermann J, McCluggage WG, McNeish I, Morice P, Pignata S, Ray-Coquard I, Vergote I, Baert T, Belaroussi I, Dashora A, Olbrecht S, Planchamp F, Querleu D; ESMO-ESGO ovarian Cancer consensus conference working group. ESMO-ESGO consensus conference recommendations on ovarian cancer: pathology and molecular biology, early and advanced stages, borderline tumours and recurrent disease†. Ann Oncol 2019;30(5):672–705. doi: 10.1093/annonc/mdz062. PMID: 31046081. 10.1093/annonc/mdz062 31046081

[12] NCCN Clinical Practice Guidelines in Oncology (NCCN Guidelines®) Ovarian Cancer Including Fallopian Tube Cancer and Primary Peritoneal Cancer Version 1. 2020 — March 11, 2020.

[13] Polterauer S, Vergote I, Concin N (2012). Prognostic Value of Residual Tumor Size in Patients With Epithelial Ovarian Cancer FIGO Stages IIa-IV: analysis of the OVCaD Data. Int J Gynecol Cancer.

[14] Bristow RE, Tomacruz RS, Armstrong DK (2002). Survival effect of maximal cytoreductive surgery for advanced ovarian carcinoma during the platinum era: a meta-analysis. J Clin Oncol.

[15] Dauplat J, Le Bouedec G, Pomel C, Scherer C (2000). Cytoreductive surgery for advanced stages of ovarian cancer. Semin Surg Oncol.

[16] Vergote I, Trope' CG, Amant F, Kristensen GB, Ehlen T, Johnson N (2010). Neoadjuvant chemotherapy or primary surgery in stage IIIC or IV ovarian cancer. N Engl J Med.

[17] Kehoe S, Hook J, Nankivell M, Jayson GC, Kitchener H, Lopes T (2015). Primary chemotherapy versus primary surgery for newly diagnosed advanced ovarian cancer (CHORUS): an openlabel, randomised, controlled, non-inferiority trial. Lancet.

[18] Onda T, Matsumoto K, Shibata T, Sato A, Fukuda H, Konishi I (2008). Phase III trial of upfront debulking surgery versus neoadjuvant chemotherapy for stage III/IV ovarian, tubal and peritoneal cancers: Japan clinical oncology group study JCOG0602. Jpn J Clin Oncol.

[19] Fagotti A, Ferrandina G, Vizzielli G, Fanfani F, Gallotta V, Chiantera V, Costantini B, Margariti PA, Gueli Alletti S, Cosentino F, Tortorella L, Scambia G. Phase III randomised clinical trial comparing primary surgery versus neoadjuvant chemotherapy in advanced epithelial ovarian cancer with high tumour load (SCORPION trial): final analysis of peri-operative outcome. Eur J Cancer 2016;59:22–33. doi: 10.1016/j.ejca.2016.01.017. Epub 2016 Mar 19. PMID: 26998845. 10.1016/j.ejca.2016.01.017 26998845

[20] Ozols RF, Bundy BN, Greer BE, Fowler JM, Clarke-Pearson D, Burger RA, Mannel RS, DeGeest K, Hartenbach EM, Baergen R; Gynecologic oncology group. Phase III trial of carboplatin and paclitaxel compared with cisplatin and paclitaxel in patients with optimally resected stage III ovarian cancer: a gynecologic oncology group study. J Clin Oncol 2003;21(17):3194–3200. doi: 10.1200/JCO.2003.02.153. Epub 2003 Jul 14. PMID: 12860964. 10.1200/JCO.2003.02.153 12860964

[21] du Bois A, Lück HJ, Meier W, Adams HP, Möbus V, Costa S, Bauknecht T, Richter B, Warm M, Schröder W, Olbricht S, Nitz U, Jackisch C, Emons G, Wagner U, Kuhn W, Pfisterer J; Arbeitsgemeinschaft Gynäkologische Onkologie ovarian Cancer study group. A randomized clinical trial of cisplatin/paclitaxel versus carboplatin/paclitaxel as first-line treatment of ovarian cancer. J Natl Cancer Inst 2003;95(17):1320–1329. doi: 10.1093/jnci/djg036. PMID: 12953086.

[22] International Collaborative Ovarian Neoplasm Group. Paclitaxel plus carboplatin versus standard chemotherapy with either single-agent carboplatin or cyclophosphamide, doxorubicin, and cisplatin in women with ovarian cancer: the ICON3 randomised trial. Lancet. 2002; 360(9332): 505–515. doi: 10.1016/S0140-6736(02)09738-6. Erratum in: Lancet. 2003 Feb 22;361(9358):706. PMID: 12241653. 10.1016/S0140-6736(02)09738-6 12241653

[23] Katsumata N, Yasuda M, Isonishi S, Takahashi F, Michimae H, Kimura E, Aoki D, Jobo T, Kodama S, Terauchi F, Sugiyama T, Ochiai K; Japanese Gynecologic Oncology Group. Long-term results of dose-dense paclitaxel and carboplatin versus conventional paclitaxel and carboplatin for treatment of advanced epithelial ovarian, fallopian tube, or primary peritoneal cancer (JGOG 3016): a randomised, controlled, open-label trial. Lancet Oncol. 2013; 14(10):1020–1026. doi: 10.1016/S1470-2045(13)70363-2. Epub 2013 Aug 13. PMID: 23948349. 10.1016/S1470-2045(13)70363-2 23948349

[24] Parmar MK, Ledermann JA, Colombo N, du Bois A, Delaloye JF, Kristensen GB, Wheeler S, Swart AM, Qian W, Torri V, Floriani I, Jayson G, Lamont A, Tropé C; ICON and AGO collaborators. Paclitaxel plus platinum-based chemotherapy versus conventional platinum-based chemotherapy in women with relapsed ovarian cancer: the ICON4/AGO-OVAR-2.2 trial. Lancet. 2003;361(9375):2099–2106. doi: 10.1016/s0140-6736(03)13718-x. PMID: 12826431. 10.1016/s0140-6736(03)13718-x 12826431

[25] Friedlander M, Trimble E, Tinker A, Alberts D, Avall-Lundqvist E, Brady M (2011). Clinical trials in recurrent ovarian cancer. Int J Gynecol Cancer.

[26] Stuart G., Kitchener H., Bacon M., Dubois A., Friedlander M., Ledermann J., et al. . (2011) 2010. Gynecologic Cancer intergroup (GCIG) consensus statement on clinical trials in ovarian cancer: report from the fourth ovarian Cancer consensus conference. Int J Gynecol Cancer 21: 750–755. 10.1097/IGC.0b013e31821b2568 21543936

[27] Oza AM, Cook AD, Pfisterer J, Embleton A, Ledermann JA, Pujade-Lauraine E, Kristensen G, Carey MS, Beale P, Cervantes A, Park-Simon TW, Rustin G, Joly F, Mirza MR, Plante M, Quinn M, Poveda A, Jayson GC, Stark D, Swart AM, Farrelly L, Kaplan R, Parmar MK, Perren TJ; ICON7 trial investigatorsStandard chemotherapy with or without bevacizumab for women with newly diagnosed ovarian cancer (ICON7): overall survival results of a phase 3 randomised trial. Lancet Oncol. 2015; 16(8): 928–936. doi: 10.1016/S1470-2045(15)00086-8. Epub 2015 Jun 23.PMID: 26115797. 10.1016/S1470-2045(15)00086-8 PMC4648090 26115797

[28] González-Martín A, Pothuri B, Vergote I, DePont Christensen R, Graybill W, Mirza MR, McCormick C, Lorusso D, Hoskins P, Freyer G, Baumann K, Jardon K, Redondo A, Moore RG, Vulsteke C, O'Cearbhaill RE, Lund B, Backes F, Barretina-Ginesta P, Haggerty AF, Rubio-Pérez MJ, Shahin MS, Mangili G, Bradley WH, Bruchim I, Sun K, Malinowska IA, Li Y, Gupta D, Monk BJ; PRIMA/ENGOTOV26/ GOG-3012 Investigators. Niraparib in Patients with Newly Diagnosed Advanced Ovarian Cancer. N Engl J Med. 2019; 381(25): 2391–2402. doi: 10.1056/NEJMoa1910962. Epub 2019 Sep 28. PMID: 31562799. 10.1056/NEJMoa1910962 31562799

[29] Moore K, Colombo N, Scambia G, Kim BG, Oaknin A, Friedlander M, Lisyanskaya A, Floquet A, Leary A, Sonke GS, Gourley C, Banerjee S, Oza A, González-Martín A, Aghajanian C, Bradley W, Mathews C, Liu J, Lowe ES, Bloomfield R, DiSilvestro P. Maintenance Olaparib in patients with newly diagnosed advanced ovarian Cancer. N Engl J Med 2018 Dec 27;379(26):2495–2505. doi: 10.1056/NEJMoa1810858. Epub 2018 Oct 21. PMID: 30345884. 10.1056/NEJMoa1810858 30345884

[30] Collinson F, Hutchinson M, Craven RA (2013). Predicting response to bevacizumab in ovarian cancer: a panel of potential biomarkers informing treatment selection. Clin Cancer Res.

[31] Backen A, Renehan AG, Clamp AR (2014). The combination of circulating Ang1 and Tie2 levels predicts progression-free survival advantage in bevacizumab-treated patients with ovarian cancer. Clin Cancer Res.

[32] Audeh MW, Carmichael J, Penson RT (2010). Oral poly (ADP-ribose) polymerase inhibitor olaparib in patients with BRCA1 or BRCA2 mutations and recurrent ovarian cancer: a proof-of-concept trial. Lancet..

[33] Gelmon KA, Tischkowitz M, Mackay H (2011). Olaparib in patients with recurrent high-grade serous or poorly differentiated ovarian carcinoma or triple-negative breast cancer: a phase 2, multicentre, open-label, non-randomised study. Lancet Oncol.

[34] Fong PC, Yap TA, Boss DS (2010). Poly (ADP)-ribose polymerase inhibition: frequent durable responses in BRCA carrier ovarian cancer correlating with platinum-free interval. J Clin Oncol.

[35] McCabe N, Turner NC, Lord CJ (2006). Deficiency in the repair of DNA damage by homologous recombination and sensitivity to poly (ADP-ribose) polymerase inhibition. Cancer Res.

[36] Coleman RL, Swisher EM, Oza AM, Scott CL, Giordano H, Lin KK, Konecny GE, Tinker A, O'Malley DM, Kristeleit RS, Ma L, Bell-McGuinn KM, Brenton JD, Cragun JM, Oaknin A, Ray-Coquard IL, Kaufmann SH, Goble S, Maloney L, Iain A (2016). McNeishRefinement of prespecified cutoff for genomic loss of heterozygosity (LOH) in ARIEL2 part 1: A phase II study of rucaparib in patients (pts) with high grade ovarian carcinoma (HGOC). J Clin Oncol.

[37] Konstantinopoulos PA, Ceccaldi R, Shapiro GI, D’Andrea AD (2015). Homologous recombination deficiency: exploiting the fundamental vulnerability of ovarian cancer. Cancer Discov.

[38] Norquist B, Wurz KA, Pennil CC, Garcia R, Gross J, Sakai W (2011). Secondary somatic mutations restoring BRCA1/2 predict chemotherapy resistance in hereditary ovarian carcinomas. J Clin Oncol.

[39] Quigley D, Alumkal JJ, Wyatt AW, Kothari V, Foye A, Lloyd P (2017). Analysis of Circulating Cell-Free DNA Identifies Multiclonal Heterogeneity of BRCA2 Reversion Mutations Associated with Resistance to PARP Inhibitors. Cancer Discov.

[40] Luvero D, Milani A, Ledermann JA. Treatment options in recurrent ovarian cancer: latest evidence and clinical potential. Ther Adv Med Oncol. 2014; 6(5): 229–239. doi: 10.1177/1758834014544121. PMID: 25342990; PMCID: PMC4206613. 10.1177/1758834014544121 PMC4206613 25342990

[41] Robert L. Coleman, Mark F. Brady, Thomas J Herzog, Deborah Kay Armstrong, Paul Sabbatini, Joan L. Walker, Byoung Kim, Keiichi Fujiwara, Krishnansu Sujata Tewari, David M. O'Malley. Bevacizumab after bevacizumab in platinum-sensitive recurrent ovarian cancer: A subgroup analysis of GOG0213. J Clin Oncol. DOI: 10.1200/JCO.2016.34.15_suppl.5523 Journal of Clinical Oncology 34, no. 15_suppl (2016) 5523.

[42] https://clinicaltrials.gov/ct2/show/NCT03106987

[43] Wu Y, Chen W, Xu ZP, Gu W. PD-L1 Distribution and Perspective for Cancer Immunotherapy-Blockade, Knockdown, or Inhibition. Front Immunol. 2019; 10: 2022. doi: 10.3389/fimmu.2019.02022. PMID: 31507611; PMCID: PMC6718566. 10.3389/fimmu.2019.02022 PMC6718566 31507611

[44] Spranger S, Gajewski TF. Impact of oncogenic pathways on evasion of antitumour immune responses. Nat Rev Cancer. 2018;18(3):139–47. 10.1038/nrc.2017.117. Epub 2018 Jan 12. 10.1038/nrc.2017.117 PMC6685071 29326431

[45] Matulonis UA, Shapira-Frommer R, Santin AD, Lisyanskaya AS, Pignata S, Vergote I, Raspagliesi F, Sonke GS, Birrer M, Provencher DM, Sehouli J, Colombo N, González-Martín A, Oaknin A, Ottevanger PB, Rudaitis V, Katchar K, Wu H, Keefe S, Ruman J, Ledermann JA (2019). Antitumor activity and safety of pembrolizumab in patients with advanced recurrent ovarian cancer: results from the phase II KEYNOTE-100 study. Ann Oncol.

[46] SGO 2019, Available on line: https://www.cancernetwork.com/view/sgo-2019-despite-missed-endpoint-javelin-trial-pd-l1-subgroup-analysis-redeems

[47] Dose Dense Paclitaxel With Pembrolizumab (MK-3475) in Platinum Resistant Ovarian Cancer - Full Text View - https://clinicaltrials.gov/ct2/show/NCT02440425.

[48] Ledermann JA, Colombo N, Oza M, et al. Avelumab in combination with and/or following chemotherapy vs chemotherapy alone in patients with previously untreated epithelial ovarian cancer: Results from the phase 3 javelin ovarian 100 trial. 2020. Society of Gynecologic Oncology Annual Meeting on Women’s Cancer. LBA 25, Scientific Plenary.

[49] Liu JF, Herold C, Gray KP, Penson RT, Horowitz N, Konstantinopoulos PA, Castro CM, Hill SJ, Curtis J, Luo W, Matulonis UA, Cannistra SA, Dizon DS. Assessment of Combined Nivolumab and Bevacizumab in Relapsed Ovarian Cancer: A Phase 2 Clinical Trial. JAMA Oncol. 2019; 5(12): 1731–1738. doi:10.1001/jamaoncol.2019.3343. Epub ahead of print. PMID: 31600397; PMCID:PMC6802049. 10.1001/jamaoncol.2019.3343 PMC6802049 31600397

[50] Moore KN, Pignata S (2019). Trials in progress: IMagyn050/GOG 3015/ENGOT-OV39. A phase III, multicenter, randomized study of atezolizumab versus placebo administered in combination with paclitaxel, carboplatin, and bevacizumab to patients with newly diagnosed stage III or stage IV o. Int. J Gynecol Cancer.

[51] Higuchi T, Flies DB, Marjon NA (2015). CTLA-4 blockade synergizes therapeutically with PARP inhibition in BRCA1-deficient ovarian Cancer. Cancer Immunol Res.

[52] Stewart RA, Pilie PG, Yap TA (2018). Development of PARP and immune-checkpoint inhibitor combinations. Cancer Res.

[53] Nguyen LT, Ohashi PS. Clinical blockade of PD1 and LAG3--potential mechanisms of action. Nat Rev Immunol 2015;15(1):45–56. doi: 10.1038/nri3790. PMID: 25534622. 10.1038/nri3790 25534622

[54] Available on line: https://clinicaltrials.gov/ct2/show/NCT03737643

[55] Available on line: https://clinicaltrials.gov/ct2/show/NCT03740165?cond=ENGOT+OV+43&draw=2&r ank=1.

[56] Available on line: https://clinicaltrials.gov/ct2/show/NCT03602859

[57] Available on line: https://clinicaltrials.gov/ct2/show/NCT03522246

[58] Available on line: https://clinicaltrials.gov/ct2/show/NCT03598270

[59] Available on line: https://clinicaltrials.gov/ct2/show/NCT02484404

[60] Available on line: https://clinicaltrials.gov/ct2/show/NCT02571725

[61] Available on line: https://www.mito-group.it/studi/studio-mito-33-nitche/

[62] Addition of a CTLA-4–Targeted Therapy to a Checkpoint Inhibitor in Ovarian Cancer - The ASCO Post. [https://www.ascopost.com/News/59329].

[63] Jihoon Kim, Bon-Kyoung Koo, Juergen A. Knoblich Human organoids: model systems for human biology and medicine Nat Rev Mol Cell Biol. 2020: 1–14. doi: 10.1038/s41580-020-0259-3 [Epub ahead of print] PMCID: PMC7339799. 10.1038/s41580-020-0259-3 PMC7339799 32636524

[64] Drost J, Clevers H. Organoids in cancer research. Nat Rev Cancer 2018;18(7):407–418. doi: 10.1038/s41568-018-0007-6. PMID: 29692415. 10.1038/s41568-018-0007-6 29692415

[65] Jacob F, Salinas RD, Zhang DY, Nguyen PTT, Schnoll JG, Wong SZH, Thokala R, Sheikh S, Saxena D, Prokop S, Liu DA, Qian X, Petrov D, Lucas T, Chen HI, Dorsey JF, Christian KM, Binder ZA, Nasrallah M, Brem S, O'Rourke DM, Ming GL, Song H. A Patient-Derived Glioblastoma Organoid Model and Biobank Recapitulates Inter- and Intra-tumoral Heterogeneity. Cell. 2020; 180(1): 188–204.e22. doi: 10.1016/j.cell.2019.11.036. Epub 2019 Dec 26.PMID: 31883794. 10.1016/j.cell.2019.11.036 PMC7556703 31883794

[66] Zhu Z, Mesci P, Bernatchez JA, Gimple RC, Wang X, Schafer ST, Wettersten HI, Beck S, Clark AE, Wu Q, Prager BC, Kim LJY, Dhanwani R, Sharma S, Garancher A, Weis SM, Mack SC, Negraes PD, Trujillo CA, Penalva LO, Feng J, Lan Z, Zhang R, Wessel AW, Dhawan S, Diamond MS, Chen CC, Wechsler-Reya RJ, Gage FH, Hu H, Siqueira-Neto JL, Muotri AR, Cheresh DA, Rich JN. Zika Virus Targets Glioblastoma Stem Cells through a SOX2-Integrin alpha(v)beta(5) Axis. Cell Stem Cell. 2020; 26(2): 187–204.e10. doi: 10.1016/j.stem.2019.11.016. Epub 2020 Jan 16.PMID: 31956038. 10.1016/j.stem.2019.11.016 PMC9628766 31956038

[67] Pauli C, Hopkins BD, Prandi D, Shaw R, Fedrizzi T, Sboner A, Sailer V, Augello M, Puca L, Rosati R (2017). Personalized in vitro and in vivo Cancer models to guide precision medicine. Cancer Discov.

[68] Phan N, Hong JJ, Tofig B, Mapua M, Elashoff D, Moatamed NA, Huang J, Memarzadeh S, Damoiseaux R, Soragni A. A simple high-throughput approach identifies actionable drug sensitivities in patient-derived tumor organoids. Commun Biol. 2019; 2: 78. doi: 10.1038/s42003-019-0305-x. PMID: 30820473; PMCID: PMC6389967. 10.1038/s42003-019-0305-x PMC6389967 30820473

[69] Maru Y, Tanaka N, Itami M, Hippo Y. Efficient use of patient-derived organoids as a preclinical model for gynecologic tumors. Gynecol Oncol. 2019;154(1):189–98. 10.1016/j.ygyno.2019.05.005. Epub 2019 May 14. 10.1016/j.ygyno.2019.05.005 31101504

[70] Hoffmann K, Berger H, Kulbe H, Thillainadarasan S, Mollenkopf HJ, Zemojtel T, Taube E, Darb-Esfahani S, Mangler M, Sehouli J, Chekerov R, Braicu EI, Meyer TF, Kessler M. Stable expansion of high-grade serous ovarian cancer organoids requires a low-Wnt environment. EMBO J. 2020; 39(6): e104013. doi: 10.15252/embj.2019104013. Epub 2020 Feb 3. PMID: 32009247; PMCID: PMC7073464. 10.15252/embj.2019104013 PMC7073464 32009247

[71] Sun H, Wang H, Wang X, Aoki Y, Wang X, Yang Y, Cheng X, Wang Z, Wang X. Aurora-A/SOX8/FOXK1 signaling axis promotes chemoresistance via suppression of cell senescence and induction of glucose metabolism in ovarian cancer organoids and cells. Theranostics. 2020; 10(15): 6928–6945. doi: 10.7150/thno.43811. PMID: 32550913; PMCID: PMC7295065. 10.7150/thno.43811 PMC7295065 32550913

[72] Nanki Y, Chiyoda T, Hirasawa A, Ookubo A, Itoh M, Ueno M, Akahane T, Kameyama K, Yamagami W, Kataoka F, Aoki D. Patient-derived ovarian cancer organoids capture the genomic profiles of primary tumours applicable for drug sensitivity and resistance testing. Sci Rep. 2020; 10(1): 12581. doi: 10.1038/s41598-020-69488-9. PMID: 32724113; PMCID: PMC7387538. 10.1038/s41598-020-69488-9 PMC7387538 32724113

[73] Chen H, Gotimer K, De Souza C, Tepper CG, Karnezis AN, Leiserowitz GS, Chien J, Smith LH. Short-term organoid culture for drug sensitivity testing of high-grade serous carcinoma. Gynecol Oncol 2020;157(3):783–792. doi: 10.1016/j.ygyno.2020.03.026. Epub 2020 Apr 4. PMID: 32253045. 10.1016/j.ygyno.2020.03.026 PMC7819712 32253045

[74] Maenhoudt N, Defraye C, Boretto M, Jan Z, Heremans R, Boeckx B, Hermans F, Arijs I, Cox B, Van Nieuwenhuysen E, Vergote I, Van Rompuy AS, Lambrechts D, Timmerman D, Vankelecom H. Developing Organoids from Ovarian Cancer as Experimental and Preclinical Models. Stem Cell Rep. 2020; 14(4): 717–729. doi: 10.1016/j.stemcr.2020.03.004. Epub 2020 Apr 2. PMID: 32243841; PMCID: PMC7160357. 10.1016/j.stemcr.2020.03.004 PMC7160357 32243841

[75] Park H, Hwang S, Jeong JY, Jung SG, Choi MC, Joo WD, Song SH, Lee C, An HJ. Integrative analysis of transcription factors and microRNAs in ovarian cancer cell spheroids. J Ovarian Res. 2020 ; 13(1): 16. doi: 10.1186/s13048-020-00618-7. PMID: 32046751; PMCID: PMC7014770. 10.1186/s13048-020-00618-7 PMC7014770 32046751

[76] Neal JT, Li X, Zhu J, Giangarra V, Grzeskowiak CL, Ju J, Liu IH, Chiou SH, Salahudeen AA, Smith AR, Deutsch BC, Liao L, Zemek AJ, Zhao F, Karlsson K, Schultz LM, Metzner TJ, Nadauld LD, Tseng YY, Alkhairy S, Oh C, Keskula P, Mendoza-Villanueva D, De La Vega FM, Kunz PL, Liao JC, Leppert JT, Sunwoo JB, Sabatti C, Boehm JS, Hahn WC, Zheng GXY, Davis MM, Kuo CJ. Organoid Modeling of the Tumor Immune Microenvironment. Cell. 2018; 175(7): 1972–1988.e16. doi: 10.1016/j.cell.2018.11.021. PMID: 30550791; PMCID: PMC6656687. 10.1016/j.cell.2018.11.021 PMC6656687 30550791

[77] Wan C, Keany MP, Dong H, Al-Alem LF, Pandya UM, Lazo S, Boehnke K, Lynch KN, Xu R, Zarrella DT, Gu S, Cejas P, Lim K, Long HW, Elias KM, Horowitz NS, Feltmate CM, Muto MG, Worley MJ, Berkowitz RS, Matulonis UA, Nucci MR, Crum CP, Rueda BR, Brown M, Liu XS, Hill SJ. Enhanced efficacy of simultaneous PD-1 and PD-L1 immune checkpoint blockade in high grade serous ovarian cancer. Cancer Res. 2020:canres.1674.2020. doi: 10.1158/0008-5472.CAN-20-1674. Epub ahead of print. PMID: 33158814. 10.1158/0008-5472.CAN-20-1674 PMC7878408 33158814

[78] Dijkstra KK, Cattaneo CM, Weeber F, Chalabi M, van de Haar J, Fanchi LF, Slagter M, van der Velden DL, Kaing S, Kelderman S, van Rooij N, van Leerdam ME, Depla A, Smit EF, Hartemink KJ, de Groot R, Wolkers MC, Sachs N, Snaebjornsson P, Monkhorst K, Haanen J, Clevers H, Schumacher TN, Voest EE. Generation of Tumor-Reactive T Cells by Co-culture of Peripheral Blood Lymphocytes and Tumor Organoids. Cell. 2018; 174(6): 1586–1598.e12. doi: 10.1016/j.cell.2018.07.009. Epub 2018 Aug 9. PMID: 30100188; PMCID: PMC6558289. 10.1016/j.cell.2018.07.009 PMC6558289 30100188

